# Oscillators and relaxation phenomena in Pleistocene climate theory

**DOI:** 10.1098/rsta.2011.0315

**Published:** 2012-03-13

**Authors:** Michel Crucifix

**Affiliations:** Georges Lemaître Centre for Earth and Climate Research, Earth and Life Institute, Université catholique de Louvain, Louvain-la-Neuve, Belgium

**Keywords:** palaeoclimates, dynamical systems, limit cycle, ice ages, Dansgaard–Oeschger events

## Abstract

Ice sheets appeared in the northern hemisphere around 3 Ma (million years) ago and glacial–interglacial cycles have paced Earth's climate since then. Superimposed on these long glacial cycles comes an intricate pattern of millennial and sub-millennial variability, including Dansgaard–Oeschger and Heinrich events. There are numerous theories about these oscillations. Here, we review a number of them in order to draw a parallel between climatic concepts and dynamical system concepts, including, in particular, the relaxation oscillator, excitability, slow–fast dynamics and homoclinic orbits. Namely, almost all theories of ice ages reviewed here feature a phenomenon of synchronization between internal climate dynamics and astronomical forcing. However, these theories differ in their bifurcation structure and this has an effect on the way the ice age phenomenon could grow 3 Ma ago. All theories on rapid events reviewed here rely on the concept of a limit cycle excited by changes in the surface freshwater balance of the ocean. The article also reviews basic effects of stochastic fluctuations on these models, including the phenomenon of phase dispersion, shortening of the limit cycle and stochastic resonance. It concludes with a more personal statement about the potential for inference with simple stochastic dynamical systems in palaeoclimate science.

## 1. Introduction

**Figure 1.**
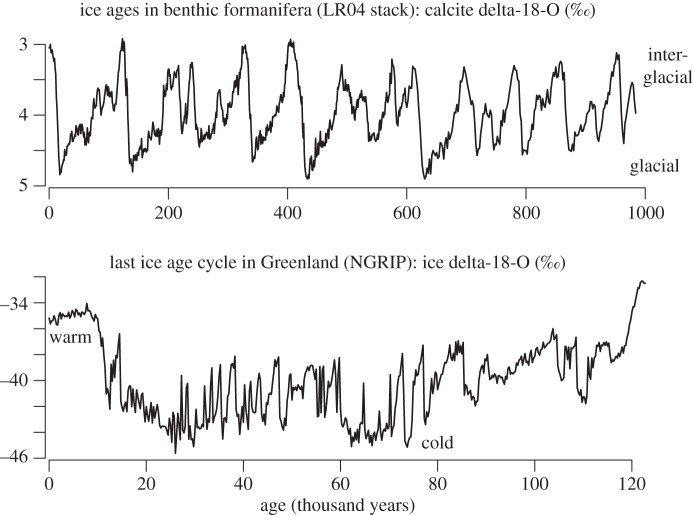
Climatic fluctuations over the Late Pleistocene. Ice ages are reconstructed using the oxygen isotopic ratio of the calcite shells of benthic foraminifera [9]. Within the last ice ages, large temperature fluctuations were recorded in Greenland, here depicted by changes in the oxygen isotopic ratio of ice [10].

The Pliocene and the Pleistocene cover approximately the past 5 Myr. The climatic fluctuations that characterized this period may be reconstructed from numerous natural archives, including marine, continental and ice core records. These archives show a complex climate history. Ice sheets appeared in the northern hemisphere around 3–3.5 Ma (million years) ago [1,2]. The volume of these ice sheets fluctuated with the variations of the seasonal and spatial distributions of incoming solar radiation (insolation), which are induced by changes in the geometry of the Earth's orbit and the angle (obliquity) between Earth's equator and the ecliptic [3–5]. This is called the astronomical forcing.^1^ Glacial cycles had an average duration of about 40 000 years [6] until about 800 000 years ago. The dominant period of glacial cycles increased around 800 000 years ago and this is referred to as the Middle Pleistocene Transition. Data and models about the Middle Pleistocene Transition are reviewed by Clark *et al*. [7]. Time-series analyses based on band-pass filtering provide further evidence of the nonlinear nature of the climate response to the astronomical forcing, from about 1.4 Ma ago [8]. The latest four glacial cycles, in particular, are distinguished by a pronounced saw-tooth time structure: ice accumulates over the continents during about 80 000 years and then melts in about 10 000 years (figure 1).

Superimposed on these long glacial cycles comes a complex pattern of millennial and sub-millennial variability [11]. For example, the Greenland record features at least 20 events of abrupt rise and slower decline in oxygen isotopic ratio (a proxy for temperature) [12,13] and methane [14] during the latest glacial epoch. These events are known as Dansgaard–Oeschger events. They were found to occur from at least the last glacial inception [15] and the Antarctic ice core records provide evidence that they are characteristic of Pleistocene glacial climates [16]. Some of these events follow pulses of iceberg discharges into the North Atlantic Ocean, called Heinrich events [17–19]. Heinrich events and Dansgaard–Oeschger events have left climatic footprints all over the globe [20], including in Antarctica [16]. The current interglacial period is referred to as the Holocene. It is also characterized by millennial and centennial variability, mainly observed in the North Atlantic [21–23], but of a much weaker amplitude than during the preceding glacial period.

This paper reviews attempts to explain these fluctuations with concepts that originate in dynamical system theory. These are the concepts of limit cycle, synchronization and excitability. The central message of the paper is that current theories of ice ages and rapid events may often be interpreted in terms of generic deterministic models, which are also used in other areas of science such as biology and ecology. However, stochastic parametrizations are an essential part of any complex system model, and their effects on climatic oscillations have to be taken into account.

Dynamical system theory entered palaeoclimate science with idealized models representing the response of ice sheets to the astronomical forcing. These models were directly derived from the physics of the ice sheet–atmosphere system [24–27]. Ghil & Childress [28], in particular, insisted on the interest of analysing such models in terms of bifurcation theory. For modelling the complex carbon cycle response, authors sometimes adopted a more heuristic approach by considering simple models and confronting the results of palaeoclimate evidence [29].

Nowadays, climate research is largely oriented towards large climate simulators (typically general circulation models), which are developed to include as many climate processes as possible. However, thinking in terms of dynamical system theory remains insightful. Indeed, the behaviour of a complex system at a certain spatio-temporal scale is in practice often dominated by a few leading modes, of which the dynamics may be captured fairly convincingly with a low-order dynamical system. Climate scientists are increasingly using this property. For example, they formulate simpler models to explain the seemingly complex behaviours observed in ocean–atmosphere simulators. Examples have been provided in recent years by focusing on interannual [30], centennial [31] and millennial [32,33] variability. In parallel, so-called hysteresis experiments, which aim at identifying the number of stable states in individual components of the climate system such as the ocean circulation [34] or ice sheets [35], contribute to a dynamical system-founded understanding of the climate system. This approach may also help us to predict and communicate about the proximity of bifurcations, which may result in catastrophic climatic changes. Timmermann & Jin [36] termed our ability to anticipate bifurcation phenomena as *predictability of the third kind*, by reference to the predictabilities of the *first* and *second* kind originally introduced by Lorenz [37].

The article is structured as follows. Section 2 reviews some of the basic concepts of oscillator theory. This is no substitute for proper textbook reading, but the reader will find essential notions and definitions needed to understand the remainder. Section 3 reviews how these concepts enter theories of ice ages and rapid events. Section 4 discusses effects of stochastic fluctuations and, finally, §5 is a more personal statement about the potential for inference with simple stochastic dynamical systems in palaeoclimate science.

## 2. Vocabulary and elementary notions

The reader will find an accessible introduction to dynamical system theory and concepts in Strogatz [38]. More formal background on oscillator theory, albeit a bit dated, is available in Guckenheimer & Holmes [39]. Bifurcation and oscillator theory is explicitly connected to climate theory in Ghil & Childress [28], in particular, ch. 12 and Saltzman [40], ch. 7. Background on synchronization and an introduction to the phenomenon of excitability is available in Pikovski *et al*. [41]. Finally, the Scholarpaedia peer-reviewed website is an increasingly rich and authoritative source of information on dynamical systems. Only the notions essential for the present article are summarized here.

*Oscillator*. The oscillator is a dynamical system that has a globally attracting limit cycle. In more simple terms, it oscillates even in the absence of an external drive. Here, we are interested in oscillators to describe climate phenomena, which involve dissipation of energy. The minimal model for a *dissipative* oscillator includes two ordinary differential equations, of which at least one is nonlinear.

**Figure 2.**
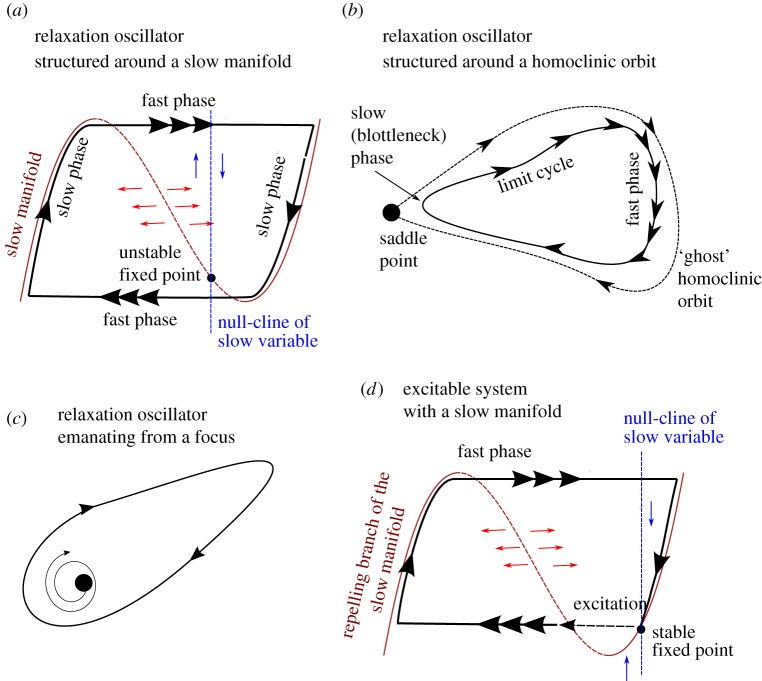
Schematic of several forms of relaxation oscillations. (*a*) The vector space is structured by a slow manifold with several stable branches. All points of the state space are attracted towards the stable branches of the slow manifold (solid lines) along the fast direction, which is here the horizontal. The slow evolution consists of an upward or downward course along the slow manifold depending on whether the system lives on the right- or left-hand side of the null-cline of the slow variable. The relaxation oscillation consists of alternate jumps between the two branches of the slow manifold. (*b*) Trajectories are rapidly attracted towards a region of the phase space influenced by a saddle point. In the scenario displayed here, there exists a combination of parameters for which the limit cycle crosses the saddle point. In that case, the period of the orbit, which includes the saddle–node, is infinitely long. It is a ‘homoclinic orbit’, hence this particular bifurcation is named a homoclinic bifurcation. The homoclinic orbit only exists at the bifurcation point, but it influences the orbit when the parameter is close to the bifurcation. This is the reason why one refers to the ‘ghost’ of the homoclinic orbit. There is another scenario for which different saddle–nodes are connected to each other. The orbit and the associated bifurcation are then said to be ‘heteroclinic’. (*c*) The relaxation oscillation is organized around a fixed point, with complex eigenvalues with a positive real part. The bifurcation giving rise to this orbit is a Hopf bifurcation. (*d*) One example of excitability, here depicted for a slow–fast system. The system resides in a stable space, but a fluctuation may cause an ejection out of the unstable (dashed lines) branch of the slow manifold. The system then loops all the way through the slow manifold before coming back to rest. (Online version in colour.)

*Relaxation oscillator*. The relaxation oscillator is a particular kind of oscillator featuring an interplay between relaxation dynamics (generally fast) and a destabilization process (generally slow). The relaxation is the process by which the system is attracted to a region of the phase space. This evokes the relaxation of a spring. In a relaxation oscillator, the system continues to evolve slowly after the relaxation phase. During this slow evolution phase, the system's stability diminishes gradually until the system is ejected out of its relaxation state, either towards another relaxation state or to the same relaxation state via a dissipative loop. In this review, we will encounter three kinds of relaxation oscillators (figure 2): relaxation founded on slow–fast dynamics (involving a slow manifold); relaxations structured by a homoclinic orbit (involving only one relaxation state); and relaxations structured around a focus. More details are provided in the caption of figure 2.

*Excitability*. An excitable system has a globally attracting fixed point (it does not oscillate spontaneously). However, an external perturbation may have the effect of *exciting* it. During this excitation, the system is being ejected far from its fixed point and then returns to it.

*Link between relaxation dynamics and excitability*. In practice, it is often found that a relaxation oscillator may be transformed into an excitable system by a mere change in parameter, and vice versa. The reason is the following. A relaxation oscillation is often structured globally in the phase space; for example, by a slow manifold (figure 2*a*) or by one or several saddle points (figure 2*b*). Suppose now that the oscillation displayed by such a system ceases because a parameter has been changed. The system is then no longer an oscillator, but the ‘backbone’ of the oscillation dynamics are still latent in the phase space because the elements that structured the limit cycle (the slow manifold or the saddle points) have not disappeared. Consequently, the system may be run on a trajectory close to the defunct limit cycle if it is being pushed by some external force (the excitation) into the region of the phase space previously occupied by this limit cycle. This point is illustrated on the basis of slow–fast dynamics in figure 2*d*, but similar excitation dynamics generally occur near any kind of ‘explosive bifurcation’, that is, bifurcations that give rise rapidly to a fully developed limit cycle. This includes homoclinic, heteroclinic and certain Hopf bifurcations (two examples follow and are illustrated in figure 6).

## 3. Oscillators, relaxation and excitability in palaeoclimates

### (a) Models of ice ages

#### (i) The Saltzman et al. models

Saltzman established a theory in which ice ages are interpreted as a limit cycle synchronized on the astronomical forcing. Saltzman *et al.* [42] wrote a series of articles on the subject, starting with the introduction of the limit cycle idea [43] and synchronization hypothesis, the interpretation of the Middle Pleistocene Transition as a bifurcation [44], and the more complete models in the mid-1990s [45]. The full theory is developed in a book [40]. Here, we concentrate on two intermediate models [46,47]. They are called SM90 and SM91, by reference to the authors (Saltzman & Maasch) and the year of publication. The variables *I*, *μ* and *θ* are the continental ice mass, CO_2_ concentration and deep-ocean temperature, respectively. The reader is referred to the original publications for the meaning and value of the different parameters. They are not crucial here; it suffices to know that they are all positive.
SM90
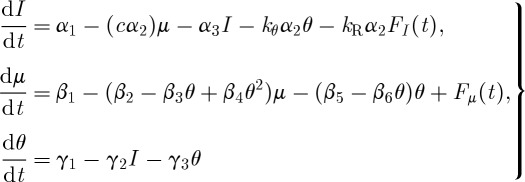

and
SM91
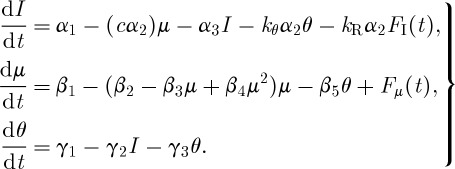



In both models, the first equation describes the ice mass response to changes in CO_2_ (*μ*) and the astronomical forcing (*F*_*I*_(*t*)); Saltzman adopts the so-called Milankovitch view^2^ that an increase in insolation causes a decrease in ice mass. Increases in CO_2_ or in ocean temperature have the same effects.

The other two equations describe the dynamics of CO_2_ and the response of deep-ocean temperature to changes in ice volume. It is further assumed that the mean state of the climate varied slowly throughout the Pliocene–Pleistocene; in particular, in response to a ‘tectonically driven’ decline in the average concentration in CO_2_, consistently with an earlier proposal [49]. This tectonically driven decline is modelled here as a slow decrease in the forcing term *F*_*μ*_(*t*) throughout the Pleistocene.

**Figure 3.**
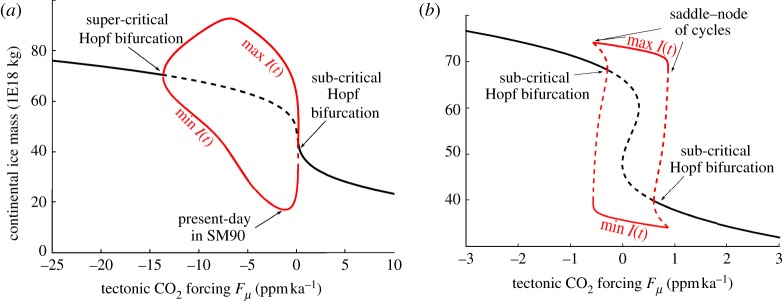
Bifurcation diagrams of the Saltzman and Maasch models (*a*) SM90 and (*b*) SM91 as a function of *F*_*μ*_, here treated as a constant control parameter. Note the difference in scales on both axes. Black lines are fixed points. Continental ice mass (*I*) is shown as a function of tectonic forcing *F*_*μ*_. The lines denoted ‘max’ and ‘min’ indicate the boundaries of the limit cycles. Unstable fixed points or limit cycles are denoted by dashed lines. Calculations and figures were made using the pseudo-arc-length continuation software package AUTO [50]. (Online version in colour.)

Consider the bifurcation diagrams of the SM90 and SM91 models with respect to *F*_*μ*_, assuming no astronomical forcing (*F*_*I*_=0) (figure 3). The systems are then said to be free or autonomous. Depending on *F*_*μ*_, both models show regimes with a stable fixed point, and regimes for which the fixed point is unstable, so that the system orbits along a limit cycle.

These considerations led Saltzman to interpret the Middle Pleistocene Transition as a bifurcation between a ‘quasi-linear’ response regime to the astronomical forcing (in the fixed-point regime) to a regime of nonlinear synchronization (resonance) on the astronomical forcing. He concluded that ice ages would occur today even in the absence of astronomical forcing. The main effect of the astronomical forcing is to control the timing of glaciations.

Saltzman's theory is seductive because it explains in a consistent framework several aspects of the Pleistocene climate history, including the change from linear to nonlinear regime [8], the presence of 100 000 year periodicity in climate records [51], the lack of a 400 000 year spectral peak in the ice volume record (such a peak appears in the simple piece-wise linear model devised by Imbrie & Imbrie [52], owing to rectification of the precession signal), the synchronization of deglaciations on the astronomical forcing [53,54], and the occurrence of large climatic transitions even when eccentricity, which modulates the effect of precession on insolation, is at its lowest.

The difficulty for accepting Saltzman's models as a definitive theory lies in the physical interpretation of the CO_2_ equation. This equation encapsulates all the interesting dynamics of the system and it is thus crucial to the theory. Some semi-empirical justification for the CO_2_ equation is given in Saltzman & Sutera [44], but the form of this equation has undergone somewhat ad hoc adjustments in SM90. The form present in SM91 is again different, with important effects on the bifurcation structure, while the authors did not justify this latter change based on physical or biogeochemical considerations.

**Figure 4.**
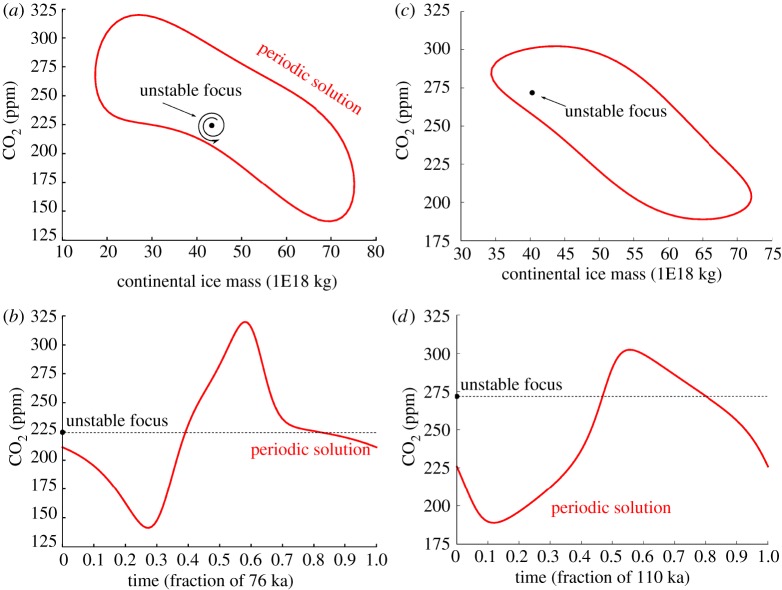
(*a*,*b*) Periodic and fixed-point solutions for SM90 (with *F*_*μ*_=1.2 ppm ka^1^) and (*c*,*d*) SM91 (with *F*_*μ*_=0.5 ppm ka^1^), near the sub-critical Hopf bifurcation points. The dynamics of SM90 slow down near the unstable fixed point, whereas the limit cycle of SM91 is much more decoupled from the position of the fixed point. (Online version in colour.)

To better appreciate the structural differences between the two models, let us return to the bifurcation diagrams. Consider SM90. As the forcing is decreased, the fixed point gives rise to a locally unstable limit cycle. The system must, therefore, find a stable limit cycle further away from the fixed point, but in this case not much further. This stable limit cycle is under the influence of the unstable fixed point and, in particular, the system slows down when it passes near it (figure 4). This scenario is depicted in figure 2*c*. The limit cycle evolves as the tectonic forcing is further decreased, until it shrinks smoothly around a perpetually glaciated state.

**Figure 5.**
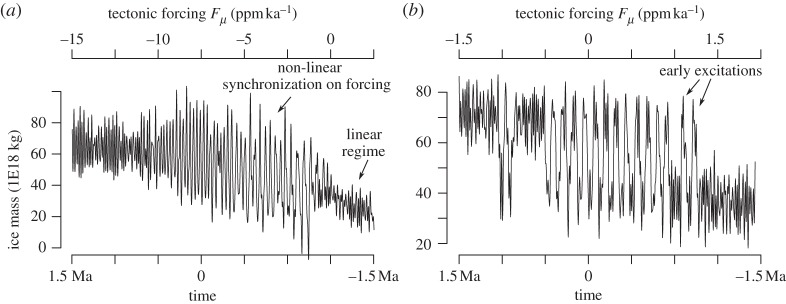
Two histories of ice volume generated with the same models (*a*) SM90 and (*b*) SM91, using the astronomical forcing and a decline scenario for the tectonic forcing of CO_2_. The scenarios were chosen here to evidence the rise and decline of 100 000 year ice ages and are not the same as those used by Saltzman. Observe the explosive character of the appearance and decline of 100 000 year cycles in SM91, with early and late excitations of the limit cycle by the astronomical forcing, and the smoother character of the evolution on SM90. Time is running from right to left.

In SM91, the bifurcation induced by the decrease in tectonic forcing is much more explosive. The system lands on a stable limit cycle that turns out to be little affected by the position of the unstable point. The cycle dynamics do not show clear phases of acceleration and the system cannot be regarded as a relaxation system. The limit cycle disappears abruptly as the tectonic forcing is further decreased, through a phenomenon called a saddle bifurcation of cycles. The consequences of the difference between the bifurcation structures of SM90 and SM91 may be further appreciated in the transient experiments shown in figure 5.

#### (ii) Paillard's (1998) ice age model (P98)

Paillard & Labeyrie [55] have been advocating the concept of relaxation for understanding palaeoclimate dynamics, both ice ages and the more abrupt events, since the publication of a seminal paper in 1994. We return to this article later on, and concentrate on another article published in 1998, in which Paillard [56] introduces a conceptual model of ice ages. Ice volume dynamics respond to an ordinary differential equation,
P98
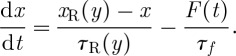

In this equation, the ice volume *x* is linearly *relaxed* to *x*_R_ with characteristic relaxation time *τ*_R_. This relaxation process is further perturbed by the astronomical forcing *F*(*t*) with a characteristic time *τ*_*f*_. Such a system is said to be hybrid [57] because the relaxation equation involves a discrete state variable, here denoted by *y*. Its state may be ‘deep glacial’ (*G*), ‘mild glacial’ (*g*) or ‘interglacial’ (*i*). The numerical values of *x*_R_ and *τ*_R_ depend on this climate state. Climate states *y* follow a sequence 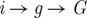 according to a set of conditions formulated on the level of glaciation *x* and insolation. Namely, the transition 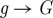 is triggered when the forcing *F*(*t*) exceeds a certain threshold. Occurrence of *G* drives climate quickly into an interglacial state *i* because *x*_R_(*G*) and *τ*_R_(*G*) are specified in the model to be low.

Paillard is not very specific about the physical meaning of the discrete variable, but it accommodates the paradigm that the Atlantic Ocean circulation has gone through three different states during the latest glacial period: intermediate circulation, shut down of the circulation and modern, deep-sinking circulation. The system (P98) features the concept of slow–fast relaxation dynamics. However, this is *not* an oscillator because the shift from *g* to *G* is determined by the course of the external forcing. The Middle Pleistocene Transition is induced in (P98) in a fashion similar to that in Saltzman, and on the basis of similar physical assumptions (tectonically driven decline in CO_2_). The drift in climatic conditions induced by tectonics is accounted for by a term added to the astronomical forcing. In a later review, Paillard [58] further emphasizes empirical evidence for the relevance of the relaxation concept in the phenomenon of deglaciation.

#### (iii) The Gildor–Tziperman model

Gildor & Tziperman [59] take a moderate step towards higher model complexity by considering a slightly more explicit representation of atmosphere, ocean, sea ice and land ice dynamics. Namely, the ocean is divided into eight boxes, and the atmosphere into four boxes. The sea ice fraction responds to standard energy balance equations. More crucially, land ice growth is influenced by a somewhat controversial feedback between sea ice and precipitation. The feedback is controversial because it is assumed that cold climate results in a *reduction* in ice volume: sea ice growth causes a reduction in precipitation in ice-covered areas and, by this mechanism, almost suppresses accumulation of snow on ice sheets. The latter then no longer compensates for ice ablation and ice volume shrinks.

A free oscillation arises from the fact that the ice volume thresholds for switching sea ice cover ‘on’ and ‘off’ differ. In other words, sea ice displays a hysteresis response to variations in ice volume. This is exactly the principle of the slow–fast relaxation oscillator depicted in figure 2*a*: the curve of equilibrium of sea ice with respect to ice volume is the slow manifold, and ice volume integrates the state of sea ice in time. In turn, this oscillation can be synchronized on the astronomical forcing.

The Gildor–Tziperman model is coupled to a biogeochemical cycle in a companion paper [60], but the essential dynamics of the glacial oscillation are unchanged. Tziperman *et al.* [61] further comment on the model and its property of synchronization on the astronomical forcing, and find that its behaviour is essentially reducible to a hybrid dynamical system.

#### (iv) The Paillard–Parrenin model

Paillard & Parrenin [62] proposed yet another relaxation model in 2004 (PP04). The prognostic variables are ice volume *I*, the area of the Antarctic continental ice sheet *A* and the atmospheric concentration in CO_2_ (*μ*) (*a*…*j* are parameters):
PP04
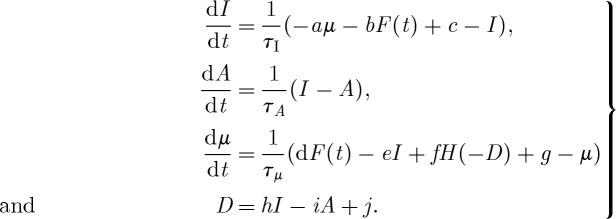



As in the other ice age models, ice volume is a slow variable driven by the astronomical forcing. It is here coupled to a variable with a similar time scale (*τ*_*A*_∼*τ*_*I*_) and a faster one (*τ*_*μ*_=*τ*_*I*_/3). The term *H*(−*D*), where *H* is the Heaviside function, represents the ventilation of the Southern Ocean. CO_2_ is released into the atmosphere when the Southern Ocean is ventilated (*D*<0), which drives deglaciation. Ice then grows slowly, until a Southern Ocean ventilation flush sends the system back to interglacial conditions. Ocean ventilation is thus the fast process in this model and it is the only nonlinear process accounted for. However, contrary to the Gildor–Tziperman model, it *does not* present a hysteresis behaviour. Consequently, the glacial cycles featured by this model cannot be interpreted in terms of shifts between the branches of a slow manifold.

**Figure 6.**
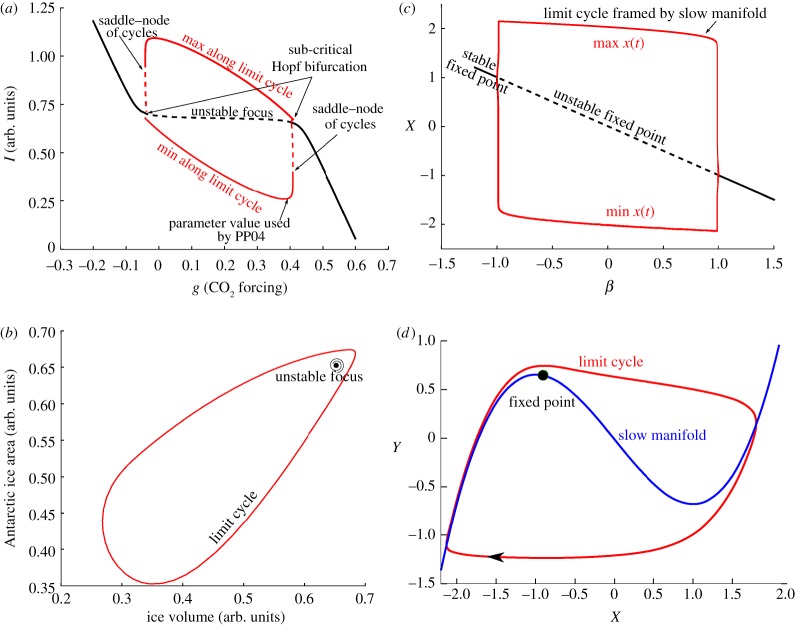
Bifurcation diagrams and phase-space trajectories of the free Paillard–Parrenin model (*a*,*b*) PP04 and (*c*,*d*) the van der Pol (VDP) model. Both systems display explosive bifurcation scenarios, but the details are different. PP04 exhibits a subcritical Hopf bifurcation, whereas the limit cycle of VDP is explicitly framed by a slow manifold. Phase-space trajectories are drawn near the bifurcation points, that is: *g*=0.4 in PP04 and *β*=0.9 in VDP. The Heaviside function in PP04 is approximated as *H*(*x*)=*a* tan(500*x*)/*π*+0.5 for analysis by AUTO. (Online version in colour.)

To better understand the dynamics of glacial cycles in this model, we consider the bifurcation diagram along typical solutions in the phase space for the free (i.e. unforced) system (figure 6). The parameter *g* is taken in this example as the control parameter, in order to preserve Saltzman's idea that ice age cycles appear as the consequence of a slow perturbation of the carbon cycle. As in SM90, PP04 exhibits a limit cycle arising from a subcritical Hopf bifurcation. The dynamics along the limit cycle close to the bifurcation point are strongly influenced by the presence of the unstable focus. This is the configuration shown in figure 2*c*. Depending on *g*, the focus is either on the low-ice-volume side of the limit cycle (i.e. the system spends most of its time with high CO_2_) or on the high-volume side of the limit cycle (i.e. the system spends most of its time in low CO_2_). Parrenin and Paillard estimate that we are currently in the second configuration.

#### (v) A minimal model of ice ages

It has been claimed [61,63] that any model that has some form of 100 000 year internal periodicity could be used to reproduce the course of ice volume over the last 800 000 years. Taking the argument at face value, Crucifix [64] used one of the simplest possible slow–fast oscillators—the van der Pol (VDP) oscillator—with minimal modifications to account for the astronomical forcing and the asymmetry between the phase of ice build-up and melt during the Late Pleistocene (*α*, *β*, *γ* and *τ* are parameters; *F*(*t*) is the astronomical forcing),
VDP
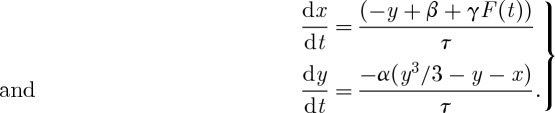



**Figure 7.**
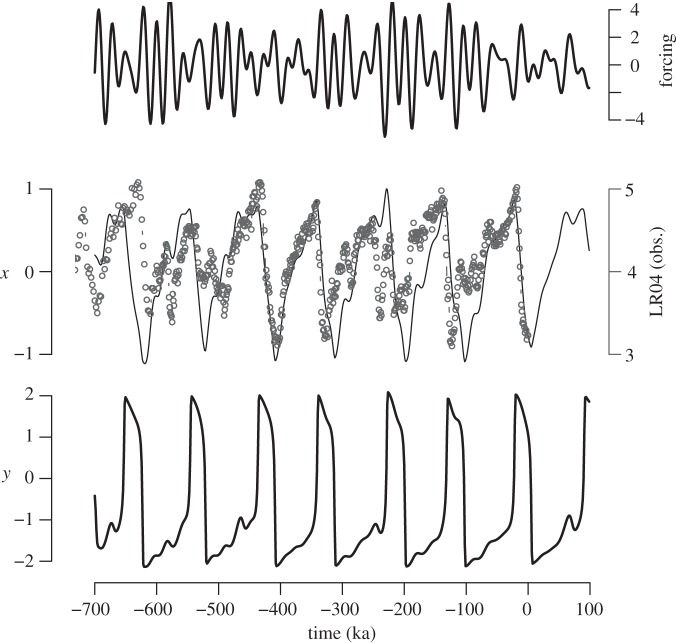
Astronomical forcing, *x* and *y* trajectories obtained using system (van der Pol, VDP) with *α*=30, *β*=0.75, *γ*=0.4 and *τ*=36 ka (1 ka=1000 years). Black dots are an authoritative natural archive thought to mainly represent fluctuations in ice volume and deep-ocean temperature, and compiled in Lisiecki & Raymo [9].

The system dynamics are determined by the structure of the slow manifold *x*=*y*^3^/3−*y*. The parameter *β* controls the position of the fixed point on the slow manifold and, consequently, the ratio of times spent by the system in the two branches (‘glacial’ and ‘interglacial’) of the slow manifold. The ice age curve can be captured with some tuning (figure 7), although it is fair to add that a small change in parameters may shift the timing of one or several ice age cycles. This minimal model was used to challenge intuitive arguments about the predictability of ice ages [64].

### (b) Models for millennial climate variability

#### (i) Dansgaard–Oeschger events as relaxation oscillations

Welander [65] introduced the concept of relaxation oscillations in the context of ocean dynamics. He described a *heat–salt oscillator* involving exchanges of heat and salt within a single oceanic column, coupled to a phenomenon of surface temperature relaxation. The destabilization process needed for the relaxation oscillation to appear is here related to diffusion between the deep ocean and the mixed layer. The system dynamics are further controlled by the mean freshwater flux at the top of the ocean column. It determines the transitions from a regime characterized by perpetual convection in the oceanic column, to a regime with intermittent convection (oscillation) and finally to a regime with no convection [66]. The bifurcations between the different regimes bear the character of *global* bifurcations, with the oscillation period approaching infinity near the bifurcation points (in particular, the second bifurcation bears the character of a homoclinic bifurcation). The heat–salt oscillator belongs thus to the class shown in figure 2*b*.

Welander [67] and Winton & Sarachik [68] later introduce the concept of another kind of relaxation oscillator in the ocean. It involves the meridional structure of the ocean thermohaline circulation, and the key nonlinear process is the meridional advection of heat and salt. The oscillations featured by this model are termed ‘deep-decoupling’ oscillations [68]. Given that the slow process now relates to heat accumulating in the global ocean, the characteristic time of deep-decoupling oscillations is of the order of 1000 years. The net flux of freshwater delivered to the North Atlantic acts as a bifurcation parameter controlling the transition between non-oscillating and oscillating regimes in the Winton–Sarachik model [69].

Millennial oscillations have since been observed across a hierarchy of ocean models, including three-dimensional ocean models with prescribed freshwater flux and restoring conditions to surface temperature [70], and three-dimensional models coupled to a simple atmosphere [71,72]. Sakai & Peltier [73] proposed that millennial deep-decoupling oscillations could explain Dansgaard–Oeschger events. Colin de Verdière *et al.* [33,74,75] complement this early proposal with a fairly complete theory based on ocean circulation model experiments. The oscillations described by Colin de Verdière *et al*. involve the processes of turbulent vertical mixing (neglected in Winton & Sarachik [68]), advection and convection, which unify the salt oscillator with the deep-decoupling oscillation model. Incidentally, Colin de Verdière *et al*. [74] dismiss the nonlinearity of the equation of state as the cause of the oscillations.

There is, across the model hierarchy, consistency about the fact that the transition between the oscillating circulation regime and the so-called diffusive, haline regime (without deep convection) is associated with a homoclinic bifurcation [33,76]. The nature of the bifurcation between the convective regime and the oscillation is more model-dependent. Timmermann *et al*. [76], based on experiments with the eight-ocean box Gildor–Tziperman model, find a Hopf bifurcation; salt-conserving experiments with a two-dimensional ocean model show a transition towards a finite-period cycle, but of increasing period as the bifurcation is approached; experiments with a more idealized model, formulated as a two-equation dynamical system, reveal the signature of an infinite-period bifurcation [33].^3^ The latter implies that Dansgaard–Oeschger events, at the time when they appear soon after the glacial inception process, should be very long but of a similar amplitude to the Dansgaard–Oeschger events coming later in the glacial cycle. This feature is consistent with the Greenland ice core record (figure 1). More specifically, the first Dansgaard–Oeschger cycles that appeared at the beginning of the glacial era were characterized by a long ‘plateau’ phase (also called interstadial) during which the thermohaline circulation was certainly very active [15]. In the Colin de Verdière *et al*. [74] theory, the plateau phase is the phase of the trajectory influenced by the ‘ghost of the saddle point’.

#### (ii) Dansgaard–Oeschger cycles as the manifestation of an excitable system

Given the explosive nature of the bifurcations involved in ocean dynamics it is no surprise to find excitability properties in ocean models. Weaver & Hughes [70] discuss this effect in salt-conserving experiments with an idealized-geometry, ocean model. The ocean–atmosphere model of intermediate complexity, CLIMBER (CLIMate BiosphERe model), was shown to exhibit excitability properties when boundary conditions are set to be typical of the latest glacial era [77]. The ocean circulation has then one stable state, with a moderate Atlantic overturning and a ‘quasi-stable state’ with more intense overturning. The conceptual sketch of the excitation cycles shown by Ganopolski & Rahmstorf [78], fig. 1 can be interpreted in terms of slow–fast dynamics, in which the different states of the ocean circulation constitute the different branches of a slow manifold. The intense overturning state, which is the ‘plateau’ phase of the Dansgaard–Oeschger event, may thus be viewed as the repelling branch of the slow manifold in the excitable regime (figure 2*d*). The excitable Dansgaard–Oeschger hypothesis was used as a possible basis to explain how a weak forcing, exogenous to the system, could explain the observed 1500 year periodicity of Dansgaard–Oeschger cycles (on this periodicity refer to earlier studies [79,80], but see the other view in the study of Ditlevsen & Ditlevsen [81]). Two such theories were developed on the basis of experiments with CLIMBER. One suggests that Dansgaard–Oeschger events are excited by stochastic fluctuations, modulated by a weak, hypothetical solar periodic forcing [82] (more on the effects of stochastic fluctuations in §4). The alternative theory suggests that the excitation is induced by the interference between two solar forcings with periods close to 1470/7(=210) and 1470/17(≈87) years [83], possibly combined with noise [84].

#### (iii) Heinrich cycles as a relaxation oscillation

MacAyeal [85] proposed an ice binge–purge theory to explain Heinrich events. The theory rests on experiments with a one-spatial direction model of ice flow dynamics. Suppose, as a starting point, that ice volume grows in response to net accumulation of snow. The growth continues until the accumulated effect of geothermal heat flux causes basal sliding. A volume of ice is then released into the ocean (this is the ‘purge’), causing the release of icebergs characteristic of Heinrich events. Ice volume thus decreases, until ice accumulation wins over so that ice volume can grow again. The ice binge–purge model is thus a relaxation oscillator combining a slow integrating process (ice mass accumulation) with a fast lateral discharge process.

#### (iv) Coupling between Heinrich and Dansgaard–Oeschger events

To what extent may Heinrich events interfere with Dansgaard–Oeschger dynamics? Paillard [55,86] investigated this question by coupling the MacAyeal ice model—but reduced to ordinary differential equations by Galerkin truncation—with a three-ocean box model. The coupling simply assumes that ice released into the ocean causes a net freshening of the surface of the North Atlantic that alters the deep-ocean circulation. Paillard realized that this coupling could lead to fairly non-intuitive and complex effects, such as the succession of Dansgaard–Oeschger events of decreasing amplitude between Heinrich events. This succession is known in the literature on palaeoclimate records as *Bond cycles* [18]. Paillard also found that the oscillations are aperiodic in this model under certain parameter configurations.

The issue is further explored in Schulz *et al.* [69], based on the Winton–Sarachik ocean model, and in Timmermann *et al*. [76], based on the slightly more sophisticated Gildor–Tziperman [59] ocean model. The objective was to study the response of deep-decoupling ocean oscillations to prescribed Heinrich cycles. Schulz *et al.* [69] noted that deep-decoupling oscillations could be synchronized on the Heinrich cycles. Timmermann *et al.* [76] then proposed, on the basis of numerical experiments with a fairly idealized model, that Heinrich events excite Dansgaard–Oeschger cycles because the variation in ice volume caused by a Heinrich event modifies slowly the amount of net freshwater released in the ocean. In turn, they suggested, Dansgaard–Oeschger may have a control on ice volume growth. This yields a two-way coupling between Dansgaard–Oeschger and Heinrich events.

Experiments with more comprehensive models of the ocean–ice sheet–atmosphere system [87,88] generally support the idea that the different water and heat fluxes involved in the different phases of ice build-up and iceberg release are quantitatively sufficient to support a coupling between ice sheets and ocean circulation during the latest glacial era. However, it was also noted that ‘three-dimensional thermomechanical ice-sheet models are unable to satisfactorily reproduce the binge–purge mechanism without an ad hoc basal parameterisation’ [89].

To address this difficulty, a theory in which Dansgaard–Oeschger events trigger Heinrich events was recently proposed [89]. The ice shelf plays a key role, in blocking the ice stream flow from the ice sheet to the oceans. Heinrich events occur when this ice shelf is broken; for example, under the influence of ocean sub-surface warming associated with a Dansgaard–Oeschger event. The resulting model is a system displaying a slow ice build-up—the Heinrich release cycle *excited* by fluctuations in ocean sub-surface temperature.

#### (v) Holocene oscillations and relationship with Dansgaard–Oeschger events

The much smaller ocean oscillations that characterized the Holocene period may also be a relaxation phenomenon. Schulz *et al.* [31] observed oscillations in the atmosphere–ocean model of intermediate complexity ECBILT-CLIO. These oscillations are related to the convective activity in the Labrador Sea.

Schulz *et al.* [90] considered the existence of such an oscillator in an earlier reference and speculated on the possible interactions between the centennial oscillations, millennial oscillations and Heinrich cycles. They considered a model in which each of these three kinds of oscillations is modelled as a Morris–Lecar relaxation oscillator. Their working hypothesis is that glacial conditions induce a coupling between these oscillators. They then observed that a very stable 1500 year oscillation appears, which they interpreted as a model equivalent of Dansgaard–Oeschger events.

## 4. Stochastic effects

The myriad of chaotic motions that characterize the dynamics of the ocean and the atmosphere may be taken into account in the form of parametrizations involving stochastic time processes. The method was introduced in climatology in the 1970s [91] and the theoretical justifications, which allow one to model chaotic motions as a (linear) stochastic process, are reviewed by Penland [92,93]. In a statistical inferential framework, the stochastic parametrizations may also be viewed as a way to account for the distance necessarily existing between the concepts and dynamics featured by the model, and the complex system being observed.

The effects of stochastic processes on relaxation oscillators and excitable systems are generally well documented in the literature because this is a topic of general interest [94]. Here, we review some of them in the specific context of palaeoclimate dynamics.

### (a) Stochastic effects on ice age dynamics

#### (i) Phase dispersion

**Figure 8.**
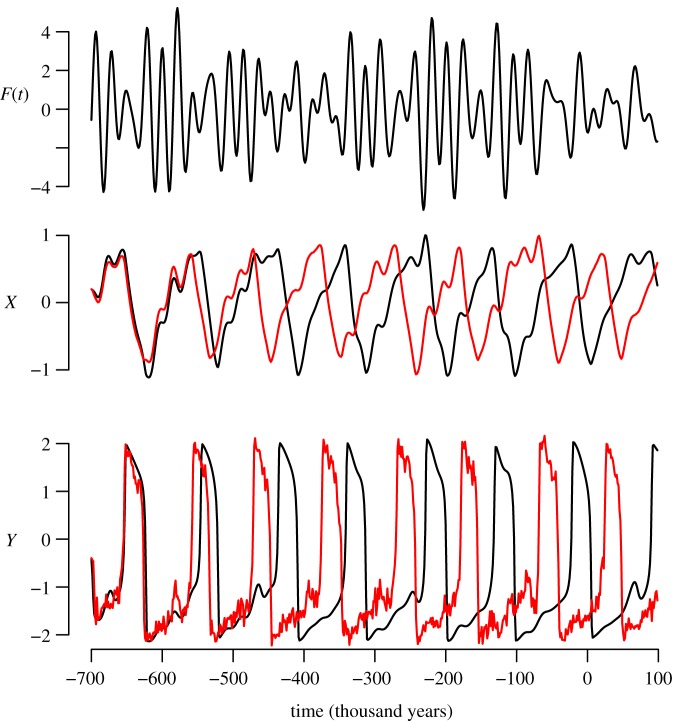
The phenomenon of trajectory shift illustrated with the model (van der Pol, VDP) with astronomical forcing, with parameters *τ*=36, *β*=0.75, *γ*= 0.4, *α*=30 ka. The black line represents the deterministic system. The generated history reproduces reasonably well the fluctuations of ice volume of the last 800 000 years. The red (grey) line represents the same system but with an additive Wiener process added to the fast variable *y*, with variance *b*=0.2 (ka)^−1/2^. One possible realization of the stochastic system is shown. Observe the solution shift around 450 000 years ago. Depending on the realization, the shift may occur at different places. (Online version in colour.)

One of the basic effects of noise on oscillators is the phenomenon of *phase dispersion*: a weak stochastic forcing on an oscillator causes a fading out of the memory of the exact initial conditions, even though the gross structure of the oscillation visualized in the phase space is conserved. The phenomenon is well known and it is an immediate consequence of the neutral stability of the phase of a free oscillator with respect to fluctuations. It was previously suggested that this phenomenon of phase dispersion may concern ice ages [95], but it is more commonly believed that ice ages are phase locked on the astronomical forcing. This phase locking should act against dispersion and permit a very long predictability horizon of ice ages. However, a phenomenon of phase dispersion may happen in oscillators that are locked on a periodic forcing. A stochastic fluctuation may momentarily cause a burst of desynchronization, called phase slip, during which the system is unhooked from its corresponding deterministic trajectory and attracted to another trajectory, which leads or lags the original one by one forcing period [41], §3.1.3. The difference between phase diffusion in a free system and in a periodic forcing-driven oscillator is that the diffusion effect has, in the latter, a quantum nature. More formally, it is said that the stochastic forcing disperses the system states around the different attractors that are compatible with the forcing. In a work in preparation, we suggest that the astronomically forced climate system may satisfy the conditions for a similar phenomenon of phase dispersion to occur (B. De Saedeleer, M. Crucifix & S. Wieczorek 2011, unpublished data). Given that the astronomical forcing is aperiodic the description of the phenomenon requires a suitable theoretical framework, which relies on the notion of a ‘local *pullback* attractor’. The equivalent of a phase slip is, in the aperiodic forcing context, a stochastic shift from one of the deterministic pullback attractors to another one. The phenomenon is illustrated based on experiments with the VDP model in figure 8.

#### (ii) Reduction of period

**Figure 9.**
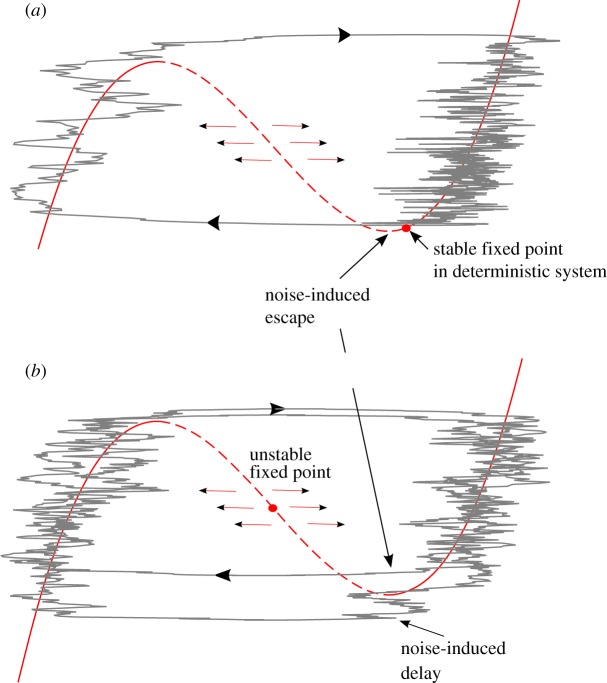
Two possible effects of additive noisy fluctuations in a slow–fast system. (*a*) If the slow–fast system is in the excitable regime, then noisy fluctuations can cause excitations. Excitations will be sporadic if the amplitude of the oscillations is weak (as shown here), or regular, as in a limit cycle, in the coherent resonance regime. (*b*) If the slow–fast system is oscillating, then additive fluctuations may cause early or delayed escapes from the slow branches, compared with the deterministic system. The net effect is a shortening of the cycle duration. (Online version in colour.)

Additive fluctuations generally reduce the period of relaxation oscillators. In an oscillator presenting a homoclinic orbit such as the Duffing oscillator, additive fluctuations reduce the time spent near the unstable focus [96]. This implies that, even at the corresponding bifurcation point in the deterministic system, the return time of oscillations in the stochastically perturbed system remains finite. In a slow–fast oscillator such as the VDP oscillator, additive fluctuations generally result in early escapes of the branch of the slow manifold on which the system lies (figure 9). The period of the oscillator is thus affected by a correction that increases approximately linearly with the noise variance in the slow–fast VDP oscillator [97].^4^ This property was used at least once in Pleistocene theory, in the silicate weathering hypothesis advanced by Toggweiler [99]. Additive fluctuations reduce the limit cycle period from 800 000 to about 100 000 years. The reasons for the period reduction being so dramatic are left for another study.

### (b) Stochastic effects on Dansgaard–Oeschger dynamics

#### (i) Stochastic excitation and resonance

Noise may naturally act as an excitation agent in an excitable system (figure 9). The topic is extensively reviewed by Lindner *et al*. [94]. Excitation loops are sporadic if the noise amplitude is weak, in which case the *recurrence time* of the events is set by the noise amplitude, whereas the *amplitude of the events* is set by the structure of the deterministic vector field. The frequency of excitation loops increases with the noise amplitude, until the system behaviour is qualitatively similar to a limit cycle regime. This is the coherent resonance regime. For yet higher noise amplitudes, the limit cycle structure is destroyed.

The concept of stochastic excitation has been considered several times in Dansgaard–Oeschger theories. The idea is introduced based on experiments with an ocean general circulation model with idealized geometry and forcing [70]. The effect of stochastic fluctuations is not only to excite oscillations in a system normally at rest, but also to reduce the period of these oscillations when the system is in oscillatory regime.

A phenomenon of non-autonomous stochastic resonance may occur if the noise is superimposed on a weak external drive. For this to happen, the autonomous system needs to be stable but excitable. The external forcing must be too weak to cause excitation by itself. The role of the noise is to provide the additional power to induce excitation. The timing of the excitation is then related to the phase of the external forcing. The mechanism was proposed several times [76,82] to explain the 1500 year recurrence time of Dansgaard–Oeschger events. The idea remains questioned, either on the grounds that the 1500 year recurrence time observed in palaeoclimate records is coincidental [81] or on the grounds that the 1500 year external forcing is unidentified [33,83]. A more subtle case of stochastic resonance involves the combination of noise with two solar cycles of 210 and 87 years, which yields the concept of ‘ghost resonance’ [100] for which some support, albeit not conclusive, is found in the observations [80].

#### (ii) Decreased sensitivity to noise in resonant oscillators

Coupled oscillators may exhibit, collectively, a resonance period that is more robust to external fluctuations than the uncoupled oscillators. Schulz *et al*. [90] used this property to explain the stability of the Dansgaard–Oeschger recurrence period of 1500 years in the presence of random fluctuations, without having to invoke an external forcing.

#### (iii) Pseudo-oscillations in two-well systems

Finally, a behaviour reminiscent of oscillations may occur in a system that is neither oscillating nor excitable, but which presents several stable states. Noise then simply induces jumps between these different states. The simplest mathematical model is the Langevin equation and this is on this basis that Schulz *et al.* [31] interpret the Holocene oscillations observed in the ECBILT-CLIO climate model.

## 5. Concluding discussion: can dynamical systems be used for inference?

The review has shown that relaxation oscillations are a popular and powerful model to explain oscillations observed in the Pleistocene record. The concept of *relaxation* implies some form of slow–fast separation, in the sense that at least one component of the system spends most of its time in ‘quasi-equilibrium’ states (this may be a ‘slow manifold branch’ or a region influenced by a saddle point, depending on the system structure), with acceleration phases.

Some of these models were constructed following a fairly careful procedure of truncation of a system of partial differential equations, which describes some of the fluid dynamics of the climate system. Others were proposed on a more conceptual basis, the idea being precisely to test a hypothesis based on palaeoclimate observations. The latter approach is sometimes criticized, on the grounds that box models, for example, cannot reasonably be taken as an adequate representation of the complex dynamics of the oceans [101].

This leads us to the last question of this review: can dynamical systems be used for inference on palaeoclimates? Inference implies that something is being learned by confronting a model with observations. This inference process may take the form of a calibration procedure (update our knowledge on parameters on the basis of observations) or a model selection procedure (which model, among different alternatives, explains the observations best).

The position taken here is that there is not such a thing as an ‘attractor’ of the climate system that is to be ‘discovered’. The hope is that some of its modes of behaviour are sufficiently decoupled from the rest of the variability to justify the fact that simple dynamical systems may capture the fundamental dynamical properties of these modes, and we want to learn about these modes from palaeoclimate observations.

The programme is challenging. Indeed, it was emphasized that different physical assumptions may lead to dynamical systems with dynamical properties that are similar enough to produce a convincing visual fit on palaeoclimate data [61]. The message is largely echoed in this review. The modeller's challenge is, therefore, to operate a model selection on more stringent criteria than just fitting some standard time series. For example, palaeoclimate observations may yield constraints on the bifurcation structure of the system. The Middle Pleistocene Transition is an attractive test case in this respect.

In a statistical inference process, the observations should be a *plausible* outcome or realization of the model. This makes sense only if the model has a stochastic component, which describes its uncertainties, limitations, and the noise that emerges from the chaotic motions of the atmosphere and oceans.

Stochastic dynamical systems are beginning to be used for inference on palaeoclimate time series. In a method called ‘potential analysis’, the climate system is modelled as a Langevin equation, that is, the combination of a down-gradient drift with a Wiener additive process and inference is made on the number of wells of the potential function [102]. The method was applied on Pleistocene climate records, yielding the conclusion that the number of wells increased from two to three over the course of the Pleistocene [103].

However, our position so far has been to favour a Bayesian methodology, because it allows one to encode physical constraints in the form of prior distributions on model parameters. The Bayesian formalism is also naturally designed for model calibration, selection and probabilistic predictions.

The fact is that Bayesian methods for selection and calibration of dynamical systems on noisy observations are only emerging. In a recent attempt, we considered a particle filter for parameter and state estimation [104]. To be honest, there is ample room for progress. Whether the process of inference with simple dynamical systems on palaeoclimate data will lead new insight in this context still needs to be demonstrated.

## Footnotes

1 The astronomical forcing will generally be taken into account here in the form of a normalized measure of insolation during the month or on the day of summer solstice at a northerly latitude, typically 60 or 65^°^ N. This is a fairly complex, aperiodic signal, with dominant harmonics corresponding to the phenomena of precession (23 716, 22 428 and 18 976 years), and obliquity (41 000 years) [5].

2 In fact, the view is introduced by Murphy [48] but it is developed mathematically in the ‘canon of insolation’ authored by Milankovitch [4].

3 This particular case is not illustrated in figure 2. There is no saddle point along or near the orbit, but there is a combination of parameters for which a fixed point appears on the limit cycle. Beyond this particular parameter value, this fixed point splits into a saddle and a node. This particular parameter value corresponds to an ‘infinite-period’ bifurcation. In practice, as long as the limit cycle exists, the trajectory slows down near the point where the saddle–node will appear. Some authors then refer to the influence of the ‘ghost’ of the saddle point [38].

4 This result is established assuming fluctuations added to the slow variable. A much more general theory, suitable for different slow manifolds and additive fluctuations to slow and fast variables, is now available [98].

## References

[1] Shackleton N. J. 1984 Oxygen isotope calibration of the onset of ice-rafting and history of glaciation in the north Atlantic region Nature 307 620 623 10.1038/307620a0 (doi:10.1038/307620a0)

[2] Meyers S. R. Hinnov L. A. 2010 Northern Hemisphere glaciation and the evolution of Plio-Pleistocene climate noise Paleoceanography 25 1 11 10.1029/2009PA001834 (doi:10.1029/2009PA001834)

[3] Croll J. (1875). Climate and time in their geological relations: a theory of secular changes of the Earth's climate.

[4] Milankovitch M. (1998). Canon of insolation and the ice-age problem.

[5] Berger A. L. 1978 Long-term variations of daily insolation and quaternary climatic changes J. Atmos. Sci. 35 2362 2367 10.1175/1520-0469(1978)035%3C2362:LTVODI%3E2.0.CO;2 (doi:10.1175/1520-0469(1978)035<2362:LTVODI>2.0.CO;2)

[6] Ruddiman W. F. Raymo M. McIntyre A. 1986 Matuyama 41 000-year cycles: North Atlantic Ocean and northern hemisphere ice sheets Earth Planet. Sci. Lett. 80 117 129 10.1016/0012-821X(86)90024-5 (doi:10.1016/0012-821X(86)90024-5)

[7] Clark P. U. Archer D. Pollard D. Blum J. D. Rial J. A. Brovkin V. Mix A. C. Pisias N. G. Roy M. 2006 The Middle Pleistocene transition: characteristics. mechanisms, and implications for long-term changes in atmospheric pCO_2_ Quat. Sci. Rev. 25 3150 3184 10.1016/j.quascirev.2006.07.008 (doi:10.1016/j.quascirev.2006.07.008)

[8] Lisiecki L. E. Raymo M. E. 2007 Plio-Pleistocene climate evolution: trends and transitions in glacial cycles dynamics Quat. Sci. Rev. 26 56 69 10.1016/j.quascirev.2006.09.005 (doi:10.1016/j.quascirev.2006.09.005)

[9] Lisiecki L. E. Raymo M. E. 2005 A Pliocene–Pleistocene stack of 57 globally distributed benthic *δ*^18^O records Paleoceanography 20 17 10.1029/2004PA001071 (doi:10.1029/2004PA001071)

[10] North Greenland Ice Core Project Members 2004 High-resolution record of northern hemisphere climate extending into the last interglacial period Nature 431 147 151 10.1038/nature02805 (doi:10.1038/nature02805) 15356621

[11] McManus J. F. Oppo D. W. Cullen J. L. 1999 A 0.5-million-year record of millennial-scale climate variability in the North Atlantic Science 283 971 975 10.1126/science.283.5404.971 (doi:10.1126/science.283.5404.971) 9974387

[12] Johnsen S. J. 1992 Irregular glacial interstadials recorded in a new Greenland ice core Nature 359 311 313 10.1038/359311a0 (doi:10.1038/359311a0)

[13] Dansgaard W. 1993 Evidence for general instability of past climate from a 250-kyr ice-core record Nature 364 218 220 10.1038/364218a0 (doi:10.1038/364218a0)

[14] Chappellaz J. Bluniert T. Raynaud D. Barnola J. M. Schwander J. Stauffert B. 1993 Synchronous changes in atmospheric CH_4_ and Greenland climate between 40 and 8 kyr BP Nature 366 443 445 10.1038/366443a0 (doi:10.1038/366443a0)

[15] Capron E. 2010 Millennial and sub-millennial scale climatic variations recorded in polar ice cores over the last glacial period Clim. Past 6 345 365 10.5194/cp-6-345-2010 (doi:10.5194/cp-6-345-2010)

[16] Loulergue L. 2008 Orbital and millennial-scale features of atmospheric CH_4_ over the past 800,000 years Nature 453 383 386 10.1038/nature06950 (doi:10.1038/nature06950) 18480822

[17] Heinrich H. 1988 Origin and consequences of cyclic ice rafting in the Northeast Atlantic Ocean during the past 130 000 years Quat. Res. 29 142 152 10.1016/0033-5894(88)90057-9 (doi:10.1016/0033-5894(88)90057-9)

[18] Bond G. 1992 Evidence for massive discharges of icebergs into the north atlantic ocean during the last glacial period Nature 360 245 249 10.1038/360245a0 (doi:10.1038/360245a0)

[19] Grousset F. E. Labeyrie L. Sinko J. A. Cremer M. Bond G. Duprat J. Cortijo E. Huon S. 1993 Patterns of ice-rafted detritus in the glacial North Atlantic (40-55N) Paleoceanography 8 175 192 10.1029/92PA02923 (doi:10.1029/92PA02923)

[20] Voelker A. H. L. 2002 Global distribution of centennial-scale records for marine isotope stage (MIS) 3: a database Quat. Sci. Rev. 21 1185 1212 10.1016/S0277-3791(01)00139-1 (doi:10.1016/S0277-3791(01)00139-1)

[21] Bond G. 1997 A pervasive millennial-scale cycle in North Atlantic Holocene and glacial climates Science 278 1257 1266 10.1126/science.278.5341.1257 (doi:10.1126/science.278.5341.1257)

[22] Bianchi G. G. McCave I. N. 1999 Holocene periodicity in north Atlantic climate and deep-ocean flow south of Iceland Nature 397 515 517 10.1038/17362 (doi:10.1038/17362)

[23] Paul A., Schulz M., Wefer G., Berger W. H., Behre K. E., Jansen E. (2002). Holocene climate variability on centennial-to-millennial time scales. I. Climate records from the North Atlantic realm. Climate development and history of the North Atlantic realm.

[24] Oerlemans J. 1980 Model experiments on the 100,000-yr glacial cycle Nature 287 430 432 10.1038/287430a0 (doi:10.1038/287430a0)

[25] Oerlemans J. (1982). Glacial cycles and ice sheet modelling. Clim. Change.

[26] Ghil M. Le Treut H. 1981 A climate model with cryodynamics and geodynamics J. Geophys. Res. 86 5262 5270 10.1029/JC086iC06p05262 (doi:10.1029/JC086iC06p05262)

[27] Le Treut H. Ghil M. 1983 Orbital forcing, climatic interactions and glaciation cycles J. Geophys. Res. 88 5167 5190 10.1029/JC088iC09p05167 (doi:10.1029/JC088iC09p05167)

[28] Ghil M., Childress S. (1987). Topics in geophysical fluid dynamics: atmospheric dynamics, dynamo theory and climate dynamics.

[29] Saltzman B. Maasch K. A. 1988 Carbon cycle instability as a cause of the late Pleistocene ice age oscillations: modeling the asymmetric response Global Biogeochem. Cycles 2 117 185 10.1029/GB002i002p00177 (doi:10.1029/GB002i002p00177)

[30] Timmermann A. 2003 Decadal ENSO amplitude modulations: a nonlinear paradigm Global Planet. Change 37 135 156 10.1016/S0921-8181(02)00194-7 (doi:10.1016/S0921-8181(02)00194-7)

[31] Schulz M. Prange M. Klocker A. 2007 Low-frequency oscillations of the Atlantic Ocean meridional overturning circulation in a coupled climate model Clim. Past 3 97 107 10.5194/cp-3-97-2007 (doi:10.5194/cp-3-97-2007)

[32] Sakai K. Peltier W. R. 1999 A dynamical systems model of the Dansgaard–Oeschger oscillation and the origin of the Bond cycle J. Clim. 12 2238 2255 10.1175/1520-0442(1999)012%3C2238:ADSMOT%3E2.0.CO;2 (doi:10.1175/1520-0442(1999)012<2238:ADSMOT>2.0.CO;2)

[33] Colin de Verdière A. 2007 A simple model of millennial oscillations of the thermohaline circulation J. Phys. Oceanogr. 37 1142 1155 10.1175/JPO3056.1 (doi:10.1175/JPO3056.1)

[34] Rahmstorf S. 2005 Thermohaline circulation hysteresis: a model intercomparison Geophys. Res. Lett. 32 L23605 10.1029/2005GL023655 (doi:10.1029/2005GL023655)

[35] Calov R. Ganopolski A. 2005 Multistability and hysteresis in the climate-cryosphere system under orbital forcing Geophys. Res. Lett. 32 L21717 10.1029/2005GL024518 (doi:10.1029/2005GL024518)

[36] Timmermann A., Jin F. F., Palmer T., Hagedorn R. (2006). Predictability of coupled processes. Predictability of weather and climate.

[37] Lorenz E. N. (1975). Climate predictability. The physical basis of climate and climate modelling.

[38] Strogatz S. H. (1994). Nonlinear dynamics and chaos, with applications to physics, biology, chemistry, and engineering (studies in nonlinearity).

[39] Guckenheimer J., Holmes P. (1983). Nonlinear oscillations, dynamical systems, and bifurcations of vector fields.

[40] Saltzman B. (2001). Dynamical paleoclimatology: generalized theory of global climate change: international geophysics.

[41] Pikovski A., Rosenblum M., Kurths J. (2001). Synchronization: a universal concept in nonlinear sciences.

[42] Saltzman B. Hansen A. R. Maasch K. A. 1984 The late Quaternary glaciations as the response of a 3-component feedback-system to Earth-orbital forcing J. Atmos. Sci. 41 3380 3389 10.1175/1520-0469(1984)041%3C3380:TLQGAT%3E2.0.CO;2 (doi:10.1175/1520-0469(1984)041<3380:TLQGAT>2.0.CO;2)

[43] Saltzman B. Sutera A. 1984 A model of the internal feedback system involved in late Quaternary climatic variations J. Atmos. Sci. 41 736 745 10.1175/1520-0469(1984)041%3C0736:AMOTIF%3E2.0.CO;2 (doi:10.1175/1520-0469(1984)041<0736:AMOTIF>2.0.CO;2)

[44] Saltzman B. Sutera A. 1987 The mid-Quaternary climatic transition as the free response of a three-variable dynamical model J. Atmos. Sci. 44 236 241 10.1175/1520-0469(1987)044%3C0236:TMQCTA%3E2.0.CO;2 (doi:10.1175/1520-0469(1987)044<0236:TMQCTA>2.0.CO;2)

[45] Saltzman B. Verbitsky M. Y. 1993 Multiple instabilities and modes of glacial rhythmicity in the Plio-Pleistocene: a general theory of late Cenozoic climatic change Clim. Dyn. 9 1 15 10.1007/BF00208010 (doi:10.1007/BF00208010)

[46] Saltzman B. Maasch K. A. 1990 A first-order global model of late Cenozoic climate Trans. R. Soc. Edinburgh Earth Sci. 81 315 325 10.1017/S0263593300020824 (doi:10.1017/S0263593300020824)

[47] Saltzman B. Maasch K. A. 1991 A first-order global model of late Cenozoic climate II. Further analysis based on a simplification of the CO_2_ dynamics Clim. Dyn. 5 201 210 10.1007/BF00210005 (doi:10.1007/BF00210005)

[48] Murphy J. J. 1876 The glacial climate and the polar ice-cap Q. J. Geol. Soc. Lond. 32 400 406 10.1144/GSL.JGS.1876.032.01-04.45 (doi:10.1144/GSL.JGS.1876.032.01-04.45)

[49] Raymo M. E. Ruddiman W. F. Froelich P. N. 1988 Influence of late Cenozoic mountain building on ocean geochemical cycles Geology 16 649 653 10.1130/0091-7613(1988)016%3C0649:IOLCMB%3E2.3.CO;2 (doi:10.1130/0091-7613(1988)016<0649:IOLCMB>2.3.CO;2)

[50] Doedel E. J., Oldeman B. E. (2009). AUTO-07P: continuation and bifurcation software for ordinary differential equations. http://indy.cs.concordia.ca/auto.

[51] Hays J. D. Imbrie J. Shackleton N. J. 1976 Variations in the Earth's orbit: pacemaker of ice ages Science 194 1121 1132 10.1126/science.194.4270.1121 (doi:10.1126/science.194.4270.1121) 17790893

[52] Imbrie J. Imbrie J. Z. 1980 Modelling the climatic response to orbital variations Science 207 943 953 10.1126/science.207.4434.943 (doi:10.1126/science.207.4434.943) 17830447

[53] Raymo M. 1997 The timing of major climate terminations Paleoceanography 12 577 585 10.1029/97PA01169 (doi:10.1029/97PA01169)

[54] Huybers P. 2007 Glacial variability over the last two million years: an extended depth-derived age model, continuous obliquity pacing, and the Pleistocene progression Quat. Sci. Rev. 26 37 55 10.1016/j.quascirev.2006.07.013 (doi:10.1016/j.quascirev.2006.07.013)

[55] Paillard D. Labeyrie L. 1994 Role of the thermohaline circulation in the abrupt warming after Heinrich events Nature 372 162 164 10.1038/372162a0 (doi:10.1038/372162a0)

[56] Paillard D. 1998 The timing of Pleistocene glaciations from a simple multiple-state climate model Nature 391 378 381 10.1038/34891 (doi:10.1038/34891)

[57] Guckenheimer J. Hoffman K. Weckesser W. 2003 The forced van der Pol equation. I. The slow flow and its bifurcations SIAM J. Appl. Dyn. Syst. 2 1 35 10.1137/S1111111102404738 (doi:10.1137/S1111111102404738)

[58] Paillard D. 2001 Glacial cycles: toward a new paradigm Rev. Geophys. 39 325 346 10.1029/2000RG000091 (doi:10.1029/2000RG000091)

[59] Gildor H. Tziperman E. 2000 Sea ice as the glacial cycles climate switch: role of seasonal and orbital forcing Paleoceanography 15 605 615 10.1029/1999PA000461 (doi:10.1029/1999PA000461)

[60] Gildor H. Tziperman E. 2001 Physical mechanisms behind biogeochemical glacial-interglacial CO_2_ variations Geophys. Res. Lett. 28 2421 2424 10.1029/2000GL012571 (doi:10.1029/2000GL012571)

[61] Tziperman E. Raymo M. E. Huybers P. Wunsch C. 2006 Consequences of pacing the Pleistocene 100 kyr ice ages by nonlinear phase locking to Milankovitch forcing Paleoceanography 21 11 10.1029/2005PA001241 (doi:10.1029/2005PA001241)

[62] Paillard D. Parrenin F. 2004 The Antarctic ice sheet and the triggering of deglaciations Earth Planet. Sci. Lett. 227 263 271 10.1016/j.epsl.2004.08.023 (doi:10.1016/j.epsl.2004.08.023)

[63] Cane M. A. 2006 Progress in paleoclimate modeling J. Clim. 19 5031 5057 10.1175/JCLI3899.1 (doi:10.1175/JCLI3899.1)

[64] Crucifix M. 2011 How can a glacial inception be predicted? Holocene 21 831 842 10.1177/0959683610394883 (doi:10.1177/0959683610394883)

[65] Welander P. 1982 A simple heat–salt oscillator Dyn. Atmos. Oceans 6 233 242 10.1016/0377-0265(82)90030-6 (doi:10.1016/0377-0265(82)90030-6)

[66] Cessi P. 1996 Convective adjustment and thermohaline excitability J. Phys. Oceanogr. 26 481 491 10.1175/1520-0485(1996)026%3C0481:CAATE%3E2.0.CO;2 (doi:10.1175/1520-0485(1996)026<0481:CAATE>2.0.CO;2)

[67] Welander P., Willebrand J., Anderson D. L. T. (1986). Thermohaline effects in the ocean circulation and related simple models. Large-scale transport processes in oceans and atmosphere.

[68] Winton M. Sarachik E. S. 1993 Thermohaline oscillations induced by strong steady salinity forcing of ocean general circulation models J. Phys. Oceanogr. 23 1389 1410 10.1175/1520-0485(1993)023%3C1389:TOIBSS%3E2.0.CO;2 (doi:10.1175/1520-0485(1993)023<1389:TOIBSS>2.0.CO;2)

[69] Schulz M. Paul A. Timmermann A. 2002 Relaxation oscillators in concert: a framework for climate change at millennial timescales during the late Pleistocene Geophys. Res. Lett. 29 2193 10.1029/2002GL016144 (doi:10.1029/2002GL016144)

[70] Weaver A. J. Hughes T. M. C. 1994 Rapid interglacial climate fluctuations driven by north Atlantic ocean circulation Nature 367 447 450 10.1038/367447a0 (doi:10.1038/367447a0)

[71] Haarsma R. J. Opsteegh J. D. Selten F. M. Wang X. 2001 Rapid transitions and ultra-low frequency behaviour in a 40 kyr integration with a coupled climate model of intermediate complexity Clim. Dyn. 17 559 570 10.1007/s003820000129 (doi:10.1007/s003820000129)

[72] Meissner K. Eby M. Weaver A. Saenko O. 2008 CO_2_ threshold for millennial-scale oscillations in the climate system: implications for global warming scenarios Clim. Dyn. 30 161 174 10.1007/s00382-007-0279-0 (doi:10.1007/s00382-007-0279-0)

[73] Sakai K. Peltier W. R. 1997 Dansgaard–Oeschger oscillations in a coupled atmosphere–ocean climate model J. Clim. 10 949 970 10.1175/1520-0442(1997)010%3C0949:DOOIAC%3E2.0.CO;2 (doi:10.1175/1520-0442(1997)010<0949:DOOIAC>2.0.CO;2)

[74] Colin de Verdière A. Ben Jelloul M. Sévellec F. 2006 Bifurcation structure of thermohaline millennial oscillations J. Clim. 19 5777 5795 10.1175/JCLI3950.1 (doi:10.1175/JCLI3950.1)

[75] Colin de Verdière A. Te Raa L. 2010 Weak oceanic heat transport as a cause of the instability of glacial climates Clim. Dyn. 35 1237 1256 10.1007/s00382-009-0675-8 (doi:10.1007/s00382-009-0675-8)

[76] Timmermann A. Gildor H. Schulz M. Tziperman E. 2003 Coherent resonant millennial-scale climate oscillations triggered by massive meltwater pulses J. Clim. 16 2569 2585 10.1175/1520-0442(2003)016%3C2569:CRMCOT%3E2.0.CO;2 (doi:10.1175/1520-0442(2003)016<2569:CRMCOT>2.0.CO;2)

[77] Ganopolski A. Rahmstorf S. 2002 Abrupt glacial climate changes due to stochastic resonance Phys. Rev. Lett. 88 038501 10.1103/PhysRevLett.88.038501 (doi:10.1103/PhysRevLett.88.038501) 11801092

[78] Ganopolski A. Rahmstorf S. 2001 Rapid changes of glacial climate simulated in a coupled climate model Nature 409 153 158 10.1038/35051500 (doi:10.1038/35051500) 11196631

[79] Schulz M. 2002 On the 1470-year pacing of Dansgaard–Oeshger warm events Paleoceanography 17 1014 10.1029/2000PA000571 (doi:10.1029/2000PA000571)

[80] Braun H. Kurths J. 2010 Were Dansgaard–Oeschger events forced by the sun? Eur. Phys. J. Spec. Top. 191 117 129 10.1140/epjst/e2010-01345-5 (doi:10.1140/epjst/e2010-01345-5)

[81] Ditlevsen P. D. Ditlevsen O. D. 2009 On the stochastic nature of the rapid climate shifts during the last ice age J. Clim. 22 446 457 10.1175/2008JCLI2430.1 (doi:10.1175/2008JCLI2430.1)

[82] Ganopolski A. Rahmstorf S. 2002 Abrupt glacial climate changes due to stochastic resonance Phys. Rev. Lett. 88 038501 10.1103/PhysRevLett.88.038501 (doi:10.1103/PhysRevLett.88.038501) 11801092

[83] Braun H. Christl M. Rahmstorf S. Ganopolski A. Mangini A. Kubatzki C. Roth K. Kromer B. 2005 Possible solar origin of the 1,470-year glacial climate cycle demonstrated in a coupled model Nature 438 208 211 10.1038/nature04121 (doi:10.1038/nature04121) 16281042

[84] Braun H. Ditlevsen P. Chialvo D. R. 2008 Solar forced Dansgaard–Oeschger events and their phase relation with solar proxies Geophys. Res. Lett. 35 5 10.1029/2008GL033414 (doi:10.1029/2008GL033414)

[85] MacAyeal D. 1993 Binge/purge oscillations of the Laurentide ice sheet as a cause of the North Atlantic's Heinrich events Paleoceanography 8 775 784 10.1029/93PA02200 (doi:10.1029/93PA02200)

[86] Paillard D. 1995 The hierarchical structure of glacial climatic oscillations: interactions between ice-sheet dynamics and climate Clim. Dyn. 11 162 177 10.1007/BF00223499 (doi:10.1007/BF00223499)

[87] Schmittner A. Yoshimori M. Weaver A. J. 2002 Instability of glacial climate in a model of the ocean–atmosphere–cryosphere system Science 295 1489 1493 10.1126/science.1066174 (doi:10.1126/science.1066174) 11823604

[88] Ganopolski A. Calov R. Claussen M. 2010 Simulation of the last glacial cycle with a coupled climate ice-sheet model of intermediate complexity Clim. Past 6 229 244 10.5194/cp-6-229-2010 (doi:10.5194/cp-6-229-2010)

[89] Alvarez-Solas J. Charbit S. Ritz C. Paillard D. Ramstein G. 2010 Links between ocean temperature and iceberg discharge during Heinrich events Nat. Geosci. 3 122 126 10.1038/NGEO752 (doi:10.1038/NGEO752)

[90] Schulz M. Paul A. Timmermann A. 2004 Glacial-interglacial contrast in climate variability at centennial-to-millennial timescales: observations and conceptual model Quat. Sci. Rev. 23 2219 2230 10.1016/j.quascirev.2004.08.014 (doi:10.1016/j.quascirev.2004.08.014)

[91] Hasselmann K. 1976 Stochastic climate models. I. Theory Tellus 28 473 485 10.1111/j.2153-3490.1976.tb00696.x (doi:10.1111/j.2153-3490.1976.tb00696.x)

[92] Penland C. 2003 Noise out of chaos and why it won't go away Bull. Am. Meteorol. Soc. 84 921 925 10.1175/BAMS-84-7-921 (doi:10.1175/BAMS-84-7-921)

[93] Penland C. Tsonis A. A. Elsner J. B. 2007 Stochastic linear models of nonlinear geosystems Nonlinear dynamics in geosciences 485 515 New York, NY Springer 10.1007/978-0-387-34918-3_27 (doi:10.1007/978-0-387-34918-3_27)

[94] Lindner B. Garcìa-Ojalvo J. Neiman A. Schimansky-Geier L. 2004 Effects of noise in excitable systems Phys. Rep. 392 321 424 10.1016/j.physrep.2003.10.015 (doi:10.1016/j.physrep.2003.10.015)

[95] Nicolis C., Nicolis C., Nicolis G. (1987). Climate predictability and dynamical systems. Irreversible phenomena and dynamical system analysis in the geosciences.

[96] Stone E. Holmes P. 1990 Random perturbations of heteroclinic attractors SIAM J. Appl. Math. 50 726 743 10.1137/0150043 (doi:10.1137/0150043)

[97] Grasman J. Roerdink J. B. T. M. 1989 Stochastic and chaotic relaxation oscillations J. Stat. Phys. 54 949 970 10.1007/BF01019783 (doi:10.1007/BF01019783)

[98] Berglund N. Gentz B. 2002 The effect of additive noise on dynamical hysteresis Nonlinearity 15 605 10.1088/0951-7715/15/3/305 (doi:10.1088/0951-7715/15/3/305)

[99] Toggweiler J. R. 2008 Origin of the 100,000-year timescale in Antarctic temperatures and atmospheric CO_2_ Paleoceanography 23 17 10.1029/2006PA001405 (doi:10.1029/2006PA001405)

[100] Braun H. Ganopolski A. Christl M. Chialvo D. R. 2007 A simple conceptual model of abrupt glacial climate events Nonlinear Process. Geophys. 14 709 721 10.5194/npg-14-709-2007 (doi:10.5194/npg-14-709-2007)

[101] Wunsch C. 2010 Towards understanding the paleocean Quat. Sci. Rev. 29 1960 1967 10.1016/j.quascirev.2010.05.020 (doi:10.1016/j.quascirev.2010.05.020)

[102] Livina V. N. Kwasniok F. Lenton T. M. 2010 Potential analysis reveals changing number of climate states during the last 60 kyr Clim. Past 6 77 82 10.5194/cp-6-77-2010 (doi:10.5194/cp-6-77-2010)

[103] Livina V. N. Kwasniok F. Lohmann G. Kantelhardt J. W. Lenton T. M. 2011 Changing climate states and stability: from Pliocene to present Clim. Dyn 37 2437 2453 10.1007/s00382-010-0980-2 (doi:10.1007/s00382-010-0980-2)

[104] Crucifix M. Rougier J. 2009 On the use of simple dynamical systems for climate predictions: a Bayesian prediction of the next glacial inception Eur. Phys. J. Spec. Top. 174 11 31 10.1140/epjst/e2009-01087-5 (doi:10.1140/epjst/e2009-01087-5)

